# Dietary omega-3 fatty acids and ionizing irradiation on human breast cancer xenograft growth and angiogenesis

**DOI:** 10.1186/1475-2867-5-12

**Published:** 2005-04-28

**Authors:** W Elaine Hardman, LuZhe Sun, Nicholas Short, Ivan L Cameron

**Affiliations:** 1 Pennington Biomedical Research Center, Louisiana State University System, Baton Rouge, Louisiana 70808 USA; 2 University of Texas Health Science Center at San Antonio, Department of Cellular and Structural Biology, San Antonio, Texas 78229 USA

## Abstract

**Background:**

The effects of an omega-3 (n-3) fatty acid enriched diet alone and in combination with gamma irradiation (IR) therapy in nude mice bearing a human MDA-MB231 breast cancer xenograft were tested. The cancer cells were injected into the mammary fat pad of young female mice. Six weeks later, mice were randomly divided into two diet groups: 1) mice with 10% corn oil (rich in omega 6 fatty acids) in their food, 2) mice consuming a 10% fat diet that was enriched in n-3 fatty acids. After two weeks on the diet, treatment with 200 cGy of IR every second day for four treatments (total 800 cGy) was initiated on half of the mice from each diet group. Some mice in each of the 4 groups were euthanized 24 hours after the end of IR while the remaining mice were followed for 3 additional weeks. Tumor sections were stained for endothelial cells with CD31 and PAS and for hypoxia inducible factor 1α (HIF-α).

**Results:**

The tumor cortex within 100 microns of the well-vascularized capsule had little vascularization. Blood vessels, capillaries, and endothelial pseudopods were found at areas greater than 100 microns from the capsule (subcortex). Mice on the corn oil diet and treated with IR 24 hours previously or non-irradiated mice fed the n-3 diet had tumors with fewer blood vessels in the subcortex and more endothelial pseudopods projecting into hypoxic (HIF- α positive) areas than did mice from the non-irradiated corn oil fed group. The tumor growth rate of mice that received IR or that were fed the n-3 fatty acid enriched diet was significantly slower than in the mice fed the 10% corn oil diet. Harmful side effects were found only in the IR treated mice.

**Conclusion:**

The omega-3 fatty acid enriched diet proved to be a safe means for retarding tumor growth and vascularization.

## Background

Animal and human epidemiological studies indicate that increasing the consumption of omega-3 (n-3) fatty acids versus intake of omega-6 (n-6) fatty acids decreased risk of cancer [1-10]. Animal studies indicate that increased consumption of n-3 fatty acids can slow the growth of cancer xenografts and can increase the efficacy and decrease the side effects of several chemotherapy agents [11-18]. Although consumption of an n-3 fatty acid enriched diet has been demonstrated to reduce tumor growth in animal xenografts, it is more likely to be used in combination with chemotherapy or with ionizing radiation (IR) therapy in the treatment of cancer patients. More than half of all cancer patients are given IR therapy during their course of treatment [19]. It therefore seemed important to determine the tumor growth retarding effects of IR and of an n-3 fatty acid enriched diet both alone and in combination. As both IR [20,21] and dietary n-3 fatty acids [22,23] are reported to suppress tumor angiogenesis, it was reasoned that the combined use of these two treatment modalities might have an additive effect on suppression of tumor growth and of angiogenesis. It was also decided to test the idea that continued consumption of n-3 fatty acids following a course of IR therapy might suppress regrowth of the IR treated tumor, perhaps by continued suppression of tumor angiogenesis.

The study reported here was therefore designed to investigate the potential of omega-3 fatty acids in the diet to inhibit growth and angiogenesis of a human breast cancer xenograft and to compare the effects of: 1) a commonly used course of IR involving exposure to 200 cGy every second day for a total of 800 cGy, 2) an n-3 fatty acid enriched diet, and 3) a combination of these two treatment regimens on tumor growth, on tumor angiogenesis, and on the side effects of each treatment regimen. Although this study used whole body IR therapy, most IR therapy of human patients is restricted to targeted regions of the body to minimize general side effects of IR treatment.

Our study results demonstrate the potential of the n-3 enriched fatty acid diet to inhibit tumor growth and angiogenesis without harmful side effects.

## Results

The four groups of mice are: 1) corn oil diet, no IR treatment, 2) corn oil diet with IR treatment, 3) n-3 enriched diet, no IR treatment, 4) n-3 enriched diet with IR treatment.

**Figure 1:**
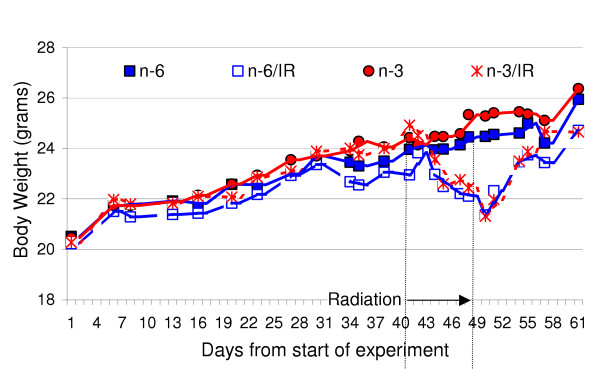
**Mean body weight of each group during the experiment. **The two groups of mice that received gamma irradiation lost body weight during and for a few days after the course of exposure, but the body weights later recovered towards the weights of the two groups of mice not exposed to gamma irradiation.

Once the mice were divided into treatment groups, the body weight of each mouse was measured every 3 to 4 days for the remainder of the experiment. As illustrated in Fig. 1, the two groups that received IR therapy every second day for 4 doses lost body weight beginning during IR therapy and lasting for about 8 or 9 days. After completion of the IR therapy, the irradiated mice again began to regain their weight toward the mean weight of the two groups of mice not subjected to IR therapy. The mean body weights of the groups of mice that did not receive IR continuously increased until the end of the experiment and were not different due to the diet.

**Figure 2:**
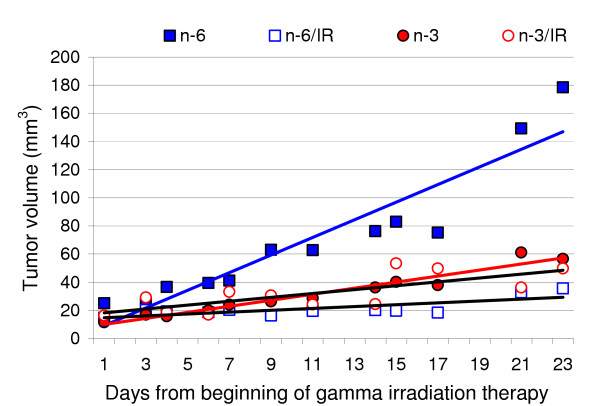
**The effect on tumor growth of consumption of a diet containing 10% corn oil (n-6) or an n-3 enriched diet (n-3) with or without gamma irradiation (IR). **The tumors were all 35 mm^3 ^or less at the start of treatment.

**Figure 3:**
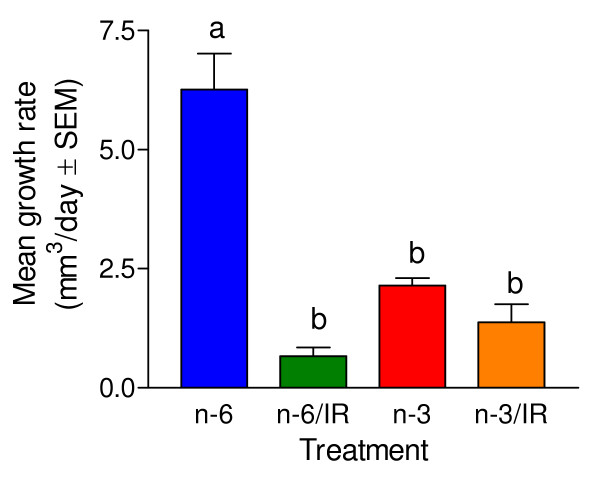
**Tumor growth rates of each treatment group**. The growth rate is the slope of the linear regression (Fig. 2) of the tumor volumes over time. Different letters demonstrate significant differences as shown by ANOVA. Compared to the group fed the n-6 diet and not irradiated, either consumption of the n-3 enriched diet or gamma irradiation significantly reduced the tumor growth.

Fig. 2 illustrates mean tumor volume for each of the four groups of mice starting at the beginning of IR therapy. All tumors in each of the four groups were less than 35 mm^3 ^and the mean tumor sizes of all four groups were similar at the start of IR treatment period. The mean growth rate of tumors from the non-irradiated mice that received the corn oil diet was significantly faster (p < 0.001) than that of the other three groups of mice (Fig. 3). The tumor growth rates of the remaining three groups of mice were not significantly different from each other.

**Figure 4:**
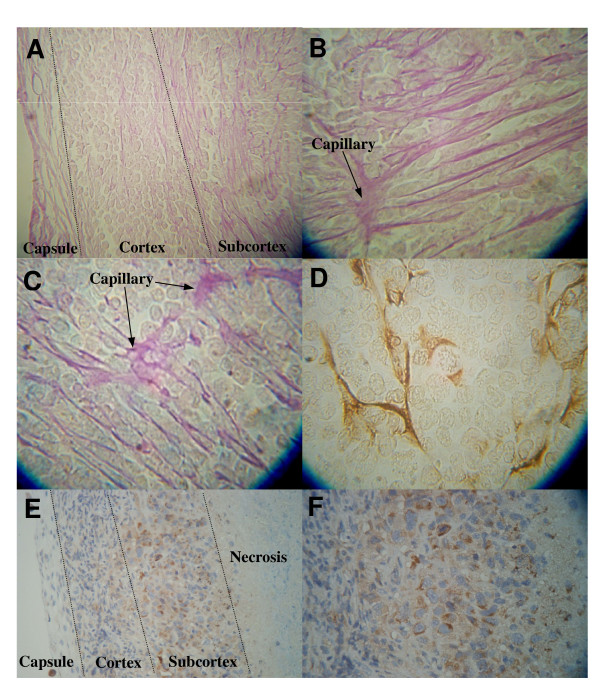
**Photomicrographs illustrating the pattern of the tumor vascular network. **A, B and C illustrate endothelial pseudopods stained with PAS, D illustrates pseudopods stained with CD-31, a specific endothelial marker and E and F illustrate the localization of HIF. (A) The tumor capsule (left) reveals blood vessels. The cortex under the capsule reveals no blood vessels and few endothelial pseudopods while the subcortical area to the right has more pseudopods. (B) The subcortical area of the tumor reveals a small blood capillary with multiple endothelial pseudopods protruding at right angles into the tumor mass. (C) At higher magnification endothelial pseudopods are seen to branch. (D) The endothelial pseudopods react positively to the CD-31 specific endothelial marker. (E) Viable cell area can be seen beneath tumor capsule (left). Necrotic area can be seen to the right. (F) Enlarged subcortical area from E. In the subcortex, the brown stain localizes HIF in the hypoxic area between the viable and the necrotic tissue.

Tumor vascularization patterns were determined from histological examination of midsections of PAS stained tumors. Tumors with a volume greater than 35 mm^3 ^demonstrated a connective tissue capsule with blood vessels (Fig. 4A). The cortical area of the tumors within about 100 μm of the capsule revealed few blood vessels while the subcortical area greater than 100 μm from the capsule showed evidence of blood vessels and capillaries with many endothelial pseudopods extending away from the capillaries (Fig. 4B&C). Immunohistochemical localization of CD-31, used as a specific marker of endothelium, demonstrated a positive reaction to blood vessels, capillaries, and pseudopods (Fig. 4D). Areas of tumor necrosis were observed below the subcortical area (Fig. 4E&F). Immunohistochemical localization of hypoxia-inducible factor 1-α (HIF-α) revealed the subcortical area of the tumor to contain HIF positive cells while the tumor capsule, cortex and necrotic areas of the tumor demonstrate no evidence of HIF. Thus, the area found to be HIF positive was enriched in endothelial pseudopods.

**Figure 5:**
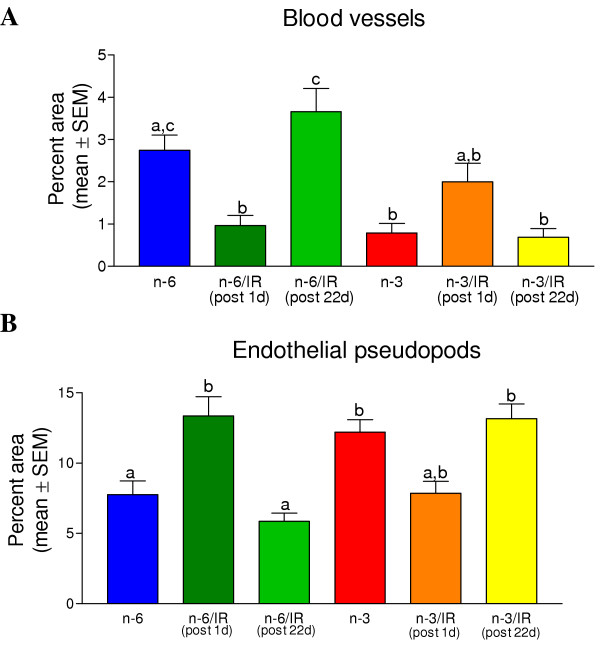
**Quantification of vascularization in the tumor subcortex of each treatment group**. The percent of areas (volume density) of blood vessels (A) and the percent of area of endothelial pseudopods (B) were determined using an ocular grid intercept counting method. The mean ± SEM of each treatment group is graphed. Columns that do not share a common letter within a graph are significantly different (p < 0.01). Data from tumors collected at the early and late times of euthanasia of non-irradiated, n-6 or n-3 fed mice were pooled because there were no significant differences between these groups due to time of euthanasia.

**Figure 6:**
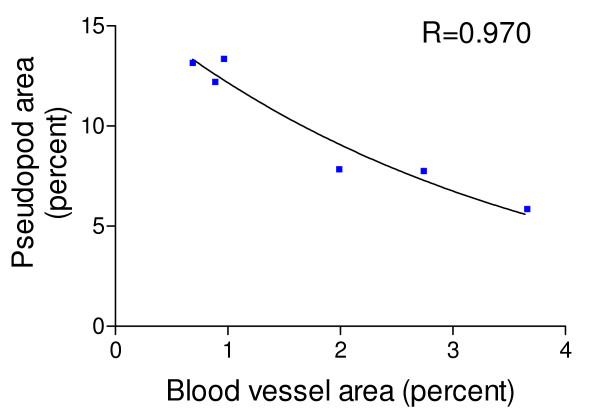
**Correlation between blood vessel and pseudopod volume density (% area) of the subcortical areas of tumors. **Data values are the mean values from Figure 5A&B. The correlation coefficient to a non-linear (logarithmic) equation is 0.970, indicating a significant inverse logarithmic fit. The pseudopod volume density decreased logarithmically as the blood vessel volume density increased.

Ocular grid intercept counting was used to quantify tumor vascularization in 8 μm thick PAS stained histological sections of the tumors. The numbers of ocular grid intercepts were scored in the subcapsular regions of the tumor over blood vessels and capillaries, endothelial pseudopods and over the area with no indication of these structures. This method has been shown to be a usable measure of volume density occupied by recognizable structures. The results of the scoring of blood vessels and of endothelial pseudopods in the subcortical regions of the tumors are summarized in Fig. 5A&B. As illustrated in Fig. 5A&B, in n-6 fed mice, the volume density of tumor blood vessels was significantly decreased but the volume density of tumor pseudopods was significantly increased at one day after the last dose of irradiation compared to the n-6 fed mice that did not receive IR. The blood vessel and pseudopods volume densities of the tumors of IR treated corn oil fed mice returned to the level of the non-irradiated corn oil fed mice by 22 days after the last dose of irradiation. However, in non-irradiated mice fed the n-3 fatty acid enriched diet, the tumor blood vessel volume density was significantly lower and the pseudopod volume density was significantly higher than in the non-irradiated n-6 fed mice. IR treatment did not significantly change the volume densities of either blood vessels or pseudopods in the tumors of n-3 fed mice. Statistical analysis of the mean blood vessel volume density versus the pseudopod volume density revealed a significant correlation (r = 0.970, Fig. 6) to an exponential fit. Thus, as the blood vessel volume density increased, the endothelial pseudopod volume density decreased.

**Figure 7:**
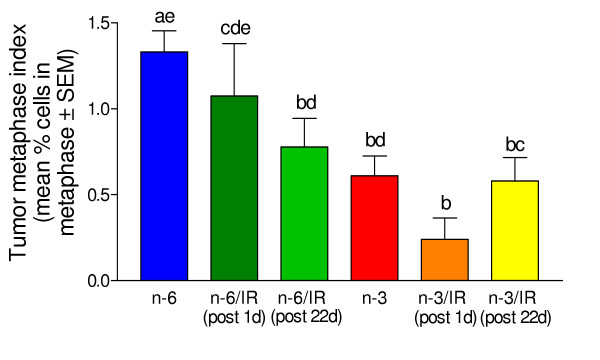
**Metaphase index (mean % of cells ± SEM) in viable areas of the tumor. **Mean values that do not share the same letter are significantly different.

Histological sections of viable areas of the tumor including both the cortex and subcortical regions were scored for number of metaphase figures. At least 1000 cancer cells were counted in each H&E stained tumor. The results are summarized in Fig. 7. The tumors of the corn oil fed group not treated with IR had the highest metaphase index. The tumors of groups of mice fed the n-3 fatty acids either with or without IR treatments had significantly fewer metaphase figures than the tumors of mice that were fed the corn oil diet but that were not treated with IR.

To assess side effects, mice were euthanized at both one day and at 22 days after the last IR exposure. Data on liver and spleen weights are summarized in Table 1, and data on blood counts are summarized in Table 2. There were no statistically significant differences in mean liver weights among treatment groups at the earlier or later kills. On the other hand, at one day after their last IR exposure, the spleen weights of the two groups of mice that received IR were significantly less than the spleen weights of the groups that did not receive IR. The mean spleen weights of the IR treated mice recovered to at least the level of the non-irradiated corn oil fed mice by 22 days after their last IR exposure.

**Table 1:** Omega-3 Fatty Acid Enriched Diet (n-3) and Gamma Irradiation Therapy (IR) on Liver and Spleen Weights (Means ± SEM)

Therapy Group	n	Liver weight (grams)	Spleen weight (grams)
Early Kill1			
n-6	5	0.94 ± 0.04	0.107 ± 0.009
n-6/IR	5	0.87 ± 0.07	0.031 ± 0.004
n-3	10	1.22 ± 0.06	0.150 ± 0.010
n-3/IR	5	1.05 ± 0.07	0.033 ± 0.003
Late Kill2			
n-6	9	1.26 ± 0.07	0.166 ± 0.008
n-6/IR	20	1.17 ± 0.03	0.146 ± 0.008
n-3	20	1.19 ± 0.04	0.166 ± 0.014
n-3/IR	13	1.20 ± 0.05	0.240 ± 0.027

^1^Mice euthanized one day after last irradiation treatment.

^2^Mice euthanized 22 days after last irradiation treatment.

**Table 2:** Omega-3 Fatty Acid Enriched Diet (n-3) and Gamma Irradiation Therapy (IR) on Blood Counts, (means ± SEM)

Therapy Group	n	WBC × 10^3^/μL	RBC × 10^6^/μL	Platelets × 10^3^/μL	Micronuclei (%RBC)
Early Kill1					
n-6	4	2.91 ± 0.83	9.08 ± 0.07	584 ± 76	1.3 ± 0.04
n-6/IR	4	0.11 ± 0.01	7.50 ± 0.16	389 ± 17	1.2 ± 0.03
n-3	7	2.02 ± 0.30	8.45 ± 0.12	634 ± 51	1.2 ± 0.03
n-3/IR	4	0.16 ± 0.03	7.66 ± 0.07	358 ± 78	1.5 ± 0.04
Late Kill2					
n-6	8	1.85 ± 0.37	8.30 ± 0.14	551 ± 83	1.6 ± 0.06
n-6/IR	13	1.48 ± 0.32	7.06 ± 0.16	973 ± 98	1.1 ± 0.01
n-3	16	2.85 ± 0.42	8.31 ± 0.20	582 ± 61	1.3 ± 0.04
n-3/IR	10	0.92 ± 0.18	6.10 ± 0.40	918 ± 131	0.9 ± 0.03

^1^Mice euthanized one day after last irradiation treatment.

^2^Mice euthanized 22 days after last irradiation treatment.

Results of Statistical Analyses:

1. At one day after the end of irradiation therapy, there were significant decreases in WBC, RBC, and platelet counts due to the irradiation. There were no other significant differences at p < 0.01.

2. At 22 days after the end of irradiation therapy, WBC and RBC counts were still significantly decreased and there was a significant increase in platelet counts due to irradiation. There were no other significant differences at p < 0.01.

There was a significant decrease in WBC, RBC, and platelets counts attributed to IR at 1 day after the end of IR treatment (Table 2). There were no significant differences in WBC, RBC, and platelets counts attributed to the n-3 fatty acid diet. At 22 days after the end of the IR treatment, the WBC and RBC counts in the two IR groups were not quite as low as they were one day post IR but remained significantly lower than that of the two non-irradiated groups. Both IR groups had significantly higher platelet counts than any of the other groups by 22 days after the end of IR therapy. Clearly, platelet counts have made a significant compensatory rebound by this time.

Micronuclei are pieces of broken chromosomes left over after the nucleus is extruded from a mature RBC and are usually an indication of genetic damage. Statistical analysis of micronuclei counts indicated no significant differences in the fraction of RBCs with micronuclei among any of the four groups at either time of euthanasia.

**Figure 8:**
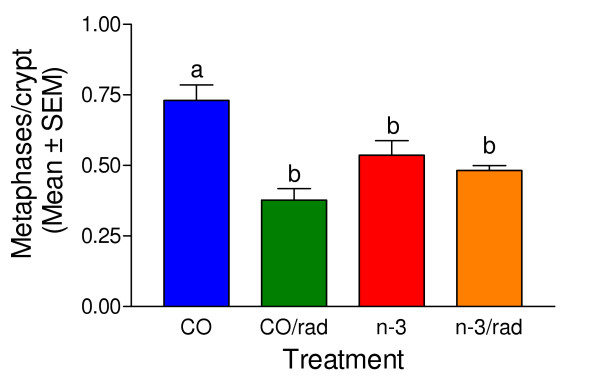
**Number of metaphase figures per midaxial histological section of duodenal crypts**. Mice were euthanized one day after the last gamma irradiation exposure. Column height indicates mean ± SEM. The columns that do not share a common letter are significantly different. The number of metaphase figures in the duodenal crypts of mice that received IR or that consumed the n-3 diet was significantly less than in the non-irradiated corn oil fed mice.

Another measure of possible side effects was the scoring of mitotic activity in the duodenal crypts. Dividing cells, such as those in duodenal crypts, are particularly sensitive to IR. Fig. 8 summarizes results of mitotic activity in the duodenal crypts of mice euthanized one day after the end of the IR therapy regimen. The metaphase index in the duodenal crypts of the IR treated corn oil fed mice was significantly lower than in the mice that did not receive IR, indicating that dividing cells were killed. However, the metaphase index in the duodenal crypts of the n-3 fed mice that were IR treated was not significantly different from that of the n-3 fed mice that were not IR treated.

## Discussion

As expected, both IR and the n-3 fatty acid enriched diet worked to suppress tumor growth. Statistical analyses of the tumor growth rate data (Fig. 3) allow comparisons of the efficacy of the four treatment procedures. The n-6 fed mice not treated with IR had the fastest tumor growth rate. The IR decreased the tumor growth rate by 90% in n-6 fed mice. Compared to the non-irradiated n-6 fed mice, the n-3 diet alone decreased the tumor growth rate by 66% whereas the combination of the n-3 and IR decreased the tumor growth rate by 78%. The tumor growth rates of the three treated groups were not significantly different. Thus, in this study, an n-3 fatty acid enriched diet alone was as effective at decreasing the growth rate of MDA-MB 231 as was the n-6 fatty acid containing diet combined with radiation. Radiation in combination with the n-3 diet was not significantly more effective than the n-3 diet alone. Since the IR treatment resulted in such good suppression of tumor growth, we cannot determine if the n-3 fatty acid diet increased the efficacy of the IR treatment or not. A different model in which IR treatment resulted in partial suppression of tumor growth would be needed to make a conclusion about the ability of n-3 fatty acids to increase the efficacy of IR therapy.

What then are the possible factors involved in the observed decrease in tumor growth rate due to the different treatment protocols? The data on blood vessel volume density indicate that either IR alone or the n-3 diet alone were equally effective at reducing the density of blood vessels in the tumors compared to that of the non-irradiated corn oil fed group. The IR treatment alone in the corn oil fed group suppressed tumor blood vessel volume density as assessed one day after the end of IR therapy. With time, the volume density of blood vessels in the tumors of the IR treated, corn oil fed group recovered to the level of that of the tumors in the corn oil fed non-irradiated group. However, the combination of the n-3 fatty acid diet and the IR continued to suppress the density of blood vessels in the tumors for 22 days after IR treatment ended.

The rapid proliferation of cancer cells requires angiogenesis to maintain oxygen and nutrient supplies to the growing tumor. Tumor cells that are far enough away (more than 100 to 150 μm) from a blood vessel can become hypoxic, which causes these tumor cells to produce HIF-α and vascular endothelial growth factor (VEGF) [21]. The VEGF then stimulates endothelial cells to sprout and produce pseudopods that project into hypoxic areas of the tumor [21]. The endothelial sprouts and pseudopods form vacuoles that fuse with capillary lumens, allowing entry of blood cells. Other steps in angiogenesis include degradation of the basement membrane surrounding existing vessels, recruitment of smooth muscle and pericyte cells, endothelial cell proliferation, lumen formation, generation of a new basement membrane, fusion of newly formed vessels and initiation of blood flow. The observed inverse relationship between the volume densities of blood vessels and of pseudopods, combined with the fact that pseudopods are most abundant in the area of the tumor most enriched in HIF-α, indicates that pseudopod volume density is an indicator of hypoxic regions in the tumor. Given this relationship, the increased pseudopod volume density as observed in tumors suggests that the subcortical areas are hypoxic. Tumor pseudopods were significantly increased one day after IR therapy in the corn oil fed mice. In the corn oil fed mice, the pseudopod volume density then decreased as the volume density of blood vessels increased compared to the non-irradiated corn oil fed mice during the 22 days recovery following the end of IR therapy. The fact that the n-3 retarded recovery of the tumor blood vessels but did not retard formation of pseudopods suggests that the n-3 diet is working to retard angiogenesis at one or more of the later steps following pseudopod formation.

There are reports that an n-3 fatty acid enriched diet can suppress mitosis and growth of breast and colon tumors [1,6,9]. Thus, it was not surprising to find that the n-3 diet also suppressed the metaphase index in viable areas of the breast cancer tumors in this study.

Together the data reveal that consumption of the n-3 containing diet resulted in a decrease in tumor growth rate, cell proliferation (Fig. 7) and blood vessel volume density (Fig. 5A). Hardman [24] has reviewed some of the possible molecular mechanisms involved in suppression of tumor growth by addition of n-3 fatty acids to the diet. The mechanisms involved in suppression of tumor growth by an omega-3 fatty acid enriched diet include: 1) decreased expression of cyclooxygenase-2, reducing angiogenesis and decreasing cancer cell proliferation, 2) suppression of nuclear factor κB activation and bcl-2 expression, allowing apoptosis of cancer cells, 3) suppression of the oncogenes AP-1 and ras, 4) induced differentiation of the cancer cells, 5) reduction in aromatase activity that decreases estrogen levels, 6) inhibition of later steps in the tumor angiogenesis process.

There has been concern that adding n-3 fatty acids to the diet might increase radiation damage to the host. N-3 fatty acids are more unsaturated than an n-6 fatty acid with the same number of carbons. The double bonds are susceptible to radiation damage and when incorporated in cellular phospholipids could increase the susceptibility of a cell to radiation induced lipid peroxidation. However, we have shown that the production of endogenous antioxidative enzymes is increased in normal cells (but not tumor cells) when n-3 fatty acids are included in the diet [18]. This increase in endogenous antioxidative enzymes could protect normal cells from oxidative damage. Damage to the bone marrow due to the n-3 diet did not seem to be increased based on either blood cell counts or numbers of micronuclei. It appears that the lower basal mitotic index in the duodenal crypts of n-3 fed mice may also have contributed to protecting the intestine from radiation damage. Irradiation is most damaging to proliferating cells. A lower basal rate of proliferation might be expected to result in less IR induced damage to cells with proliferative capacity. The metaphase index of the duodenum crypts of n-3 fed mice after IR is much closer to that of the non-irradiated mice than in the n-6 fed mice after IR, indicating either faster recovery or less initial killing by IR of proliferative cells in the duodenum in the n-3 fed mice.

The whole body radiation of mice was expected to demonstrate significant side effects within a day following the eight-day course of IR therapy. The observed side effects included significant decreases in WBC, RBC, and platelet counts as well as loss of spleen weight. These measurable side effects serve as reference for comparison with any side effects of the n-3 enriched diet alone or in combination with IR therapy. In this study, there was no evidence of increased side effects due to the n-3 diet nor did it seem to significantly lessen the measured side effects of IR therapy. This failure of the n-3 diet to lessen the side effects of IR therapy differs from past reports that omega-3 fatty acid supplements ameliorated side effects of several chemotherapeutic agents [11-15]. Most of the observed side effects with whole body irradiation are limited in human patients by targeting of IR to tumorous regions.

The n-3 diet alone significantly suppressed tumor growth. However, in the short term, the efficacy of the IR therapy was not significantly greater in mice that consumed the n-3 enriched diet than in the mice that consumed the corn oil diet. Others have reported that the efficacy of radiation therapy was increased in the presence of an n-3 containing diet [25]. It is speculated that the n-3 oil diet reduced blood vessel volume density, causing increased areas of the tumor to become hypoxic, thus rendering these hypoxic areas of the tumor less sensitive to the oxidative damage of IR. Had our experiment continued longer than three weeks after the radiation, the results might well have been different. Angiogenesis suppression by the n-3 diet would have continued to suppress growth of residual tumors in the n-3 fed mice but residual tumors in the n-6 fed mice were stimulating angiogenesis and could have resumed growth. In support of this idea, analyses of tumor growth in the IR treated n-3 fed and n-6 fed mice beginning 7 days after the end of radiation indicates that there may be a resumption of growth of the residual tumor in the n-6 fed mice but not in the n-3 fed mice. The tumor growth rate (mean ± SD) from linear regression analyses of the last four available data points of the n-6 fed, IR treated mice is 2.3 ± 0.5 mm^3^/day, a significant (p 60; 0.05) positive growth rate. However, the tumor growth rate (mean ± SEM) of the n-3 fed, IR treated mice for the last four data points is -1.0 ± 1.0 mm^3^/day, a rate that is not significantly (p = 0.5) different from 0. Due to the few points, these tumor growth rates are not quite significantly different (p = 0.07) yet the data does suggest that in a longer term study, n-3 may provide a better treatment advantage than is indicated by the results of this study.

## Conclusion

In conclusion, an omega-3 fatty acid enriched diet was found to significantly reduce the growth rate and angiogenesis of a human breast cancer xenograft without evidence of harmful side effects.

## Methods

All animal use and care procedures were approved by the Institutional Animal Care and Use Committees of the University of Texas Health Science Center at San Antonio and of the Pennington Biomedical Research Center. The human breast cancer cell line MDA-MB-231 was obtained from the American Type Culture Collection. The cell lines were cultured in McCoys's 5A medium supplemented with pyruvate, vitamins, amino acids, antibiotics, and 10% fetal bovine serum [26]. The MDA-MB-231 cells were harvested from exponential cultures and inoculated at 2 × 10^6^cells/inoculum in the inguinal mammary fat pad area of 160 female athymic nude mice, 6 weeks of age (purchased from the Harlan Sprague Dawley, Inc., Indianapolis, IN). The animals were housed under pathogen-free conditions and fed an AIN-76 semipurified diet slightly altered to contain 10% w/w corn oil during the initial tumor growth period. Growth of each xenograft was monitored three times per week by externally measuring tumors in three dimensions using digital calipers. Xenograft volume (V) was determined by the following equation: V = (L × W × D) × 0.5, where L is the length, W is the width and D is the depth of a xenograft. When the tumors reached an average diameter of about 3 mm after 5 weeks, the mice were divided into 2 groups such that the mean and median of tumor volume of the groups were closely matched.

Half of the mice fed the 10% w/w corn oil diet for the remainder of the study, half of the mice were placed on the experimental diet that contained 5% w/w n-3 fatty acids supplement (AAFA, Incell, Corp., LLC, San Antonio, TX) and 5% w/w canola oil. Corn oil is 61% linoleic acid (18:2n-6) and about 1% n-3 fatty acid thus it provides primarily n-6 FAs. The 5% w/w canola oil was used as the source of essential n-6 FAs in the n-3 diet. Canola oil is 20% linoleic acid and it also contains about 10% alpha-linolenic acid (18:3n-3). Five percent w/w n-3 FAs supplement (AAFA) containing 6% n-6 fatty acid and 61% total n-3 fatty acids was added to greatly increase the n-3 content of this diet. The total w/w composition of the n-6 diet was 0.1% n-3 FAs and 6.1% n-6 FAs with an n-3/n-6 ratio of 0.1. The total w/w composition of the n-3 diet was 3.65% n-3 FAs and 1.3% n-6 FAs with an n-3/n-6 FAs ratio of 2.8:1. These diets provided large differences in the fatty acids thought to be associated with cancer growth, linoleic acid and n-3 FAs.

Half of the tumor-bearing mice of each diet group received a cumulative dose of 800 cGy of IR (200 cGy each second day for 4 cycles). The 200 cGy/day dosage is based on the results from a preliminary dose response study using the same mouse model and on the fact that 180 to 250 cGy/day is the commonly used acute dose for radiotherapy of humans. About 815 cGy whole body radiation is the LD50/30 for mice (50% lethal within 30 days). Mice were transferred to a circular cage with individual compartments for each mouse during irradiation. Mice were irradiated in a ^137^Cs Gamma Cell-40 Irradiator (Atomic Energy of Canada) facility in our Department of Radiology. A dosimetric analysis for this instrument is performed monthly for calculation of a precise 200 cGy exposure. Mice were euthanized at one day or 22 days after the last radiation treatment.

Mice were deeply anesthetized using a ketamine/rompun mixture prepared by the UTHSCSA Laboratory Animal veterinarian, cervically dislocated, then were exsanguinated by cardiac puncture. Blood was collected into an EDTA containing microtube for complete blood counts.

The tumor, duodenum, spleen and liver were removed at the time of euthanasia. Samples of the duodenum and the tumor were fixed in Omni Fix II (Mt. Vernon N.Y.) and paraffin embedded. Embedded tissues were cut 4 μm or 8 μm thick, cut sections were placed on microscope slides then deparaffinized and stained with hematoxylin and eosin or with periodic acid-Schiff (PAS) for morphological analysis. Additional sections were prepared for immunohistochemistry.

To determine the effect of treatment on tumor angiogenesis, we measured the vascularity of excised tumors. Tumor tissues were fixed and embedded in paraffin. Mid tumor sections (8 μm thick) were cut from the embedded tissue and stained with periodic acid Schiff (PAS). Sections were examined by light microscopy. CD31 immunostaining for mouse blood vessels on 4 μm thick sections was performed by incubating tumor sections with a rat antimouse CD-31 (PECAM-1) monoclonal antibody (PharMingen) at 5 μg/ml for 30 min at 37°C. Sections were then incubated with a biotin-labeled goat anti-rat IgG (Zymed; 1:200 dilution) for 30 min at room temperature, followed by ABC reagent kit (Vector Laboratories) for 30 min at room temperature. Color reaction was performed with 3, 3'-diaminobenzidine (Vector Laboratories) and counterstained with hematoxylin. Hypoxia-inducible factor-1 alpha (HIF-1α) immunohistochemistry was done following the instruction for antigen retrieval (Biogenex protocol) and iso-IHC (inno-Genex Mouse-on-Mouse iso-IHC kit) with these changes: Dewaxing was with 3 changes of xylene, 10 minutes/change. Rehydration in 100%, 90%, and 70% ethanol for 10 minutes each. Initial dilution of the antibody was 1:200. Stressgen anti-HIF-1 alpha, product # OSA-601, was the antibody used. All sections were coded, treated as above and the extent of blood vessels, endothelial cell pseudopods and total area volume density was scored using an ocular grid. The number of grid line intercepts over blood vessels and endothelial pseudopods gave a measure of the total volume density of these structures.

A blood cell counter with veterinary pack was used for counts of red cells, white cells and platelets in EDTA anticoagulated blood of the mice. Our Laboratory Animal Resources division performed this test. Micronuclei counts were determined on thin smears of whole blood. Blood smears were stained with 0.1% acridine orange in phosphate buffered saline (pH 7.4) for 10 seconds. Slides were then examined for the presence of micronuclei using a fluorescent microscope and a 100X oil immersion objective. At least 1000 acridine orange stained red blood cells were counted and the percentage of erythrocytes containing micronuclei was determined [27].

Fixed specimens of duodenum were trimmed, processed and oriented for paraffin embedding. Four μm thick sections of the paraffin blocks were mounted on slides. Complete midaxially sectioned crypts on H&E stained slides were selected for analyses. Complete crypts were defined as those with: 1) the crypt base at the muscularis mucosa, 2) an open lumen from mouth to base and 3) a single column of epithelial cells up each side of the crypt. The numbers of metaphase figures per midaxial crypt section were counted for 10 crypts of each mouse.

SAS computer software was used for statistical analyses of numerical data. Tests for normality (basic statistics) were used on each data set. One-way and two-way analyses of variance followed by Student-Newman-Keuls multiple range tests, as appropriate, was used to determine if there were statistically significant (p ≤ 0.05) differences in any measured parameter due to the therapies. Prism™ (Graphpad Software, Inc.) was used for statistical analyses of tumor growth data. The mean growth rate for each group was determined using least squares linear regression analysis of mean tumor volume with time. Analyses of variance of the slopes of the linear regression (growth rate) were used to determine statistical differences in mean growth rates between treatment groups.

## Competing interests

WEH and ILC are scientific advisors for Incell, Corp., LLC. They do not receive financial compensation from and no research support was received from Incell, Corp., LLC.

## Authors' contributions

WEH and ILC contributed equally to writing this manuscript. LZS provided the tumor cells, NS assisted with daily animal care and graph preparation. All authors have read and approved this manuscript.
